# Stress, mental health and sociocultural adjustment in third culture kids: exploring the mediating roles of resilience and family functioning

**DOI:** 10.3389/fpsyg.2023.1093046

**Published:** 2023-08-14

**Authors:** Emma E. Jones, Marnie Reed, Andrea H. Meyer, Jens Gaab, Yoon P. Ooi

**Affiliations:** ^1^University of Basel, Basel, Switzerland; ^2^Institute of Mental Health, Singapore, Singapore

**Keywords:** adjustment, stress, family functioning, third culture kid, resilience

## Abstract

**Introduction:**

This cross-sectional study explores the contributions of personal and contextual factors in the adjustment process of a sample of internationally mobile children and adolescents having relocated to Switzerland. Based on evolutionary developmental theories and recommendations by Research Domain Criteria and The Hierarchical Taxonomy of Psychopathology theoretical frameworks, we hypothesized and tested a heuristic model of TCK adjustment, aiming to identify prevention and treatment targets tailored for our sampled population.

**Methods:**

We assessed the relationships in the hypothesized models, particularly how perceived and acculturative stress influence TCK adjustment and whether the relationship between the predictors of TCK stress and the outcomes of TCK adjustment are mediated by resilience and family functioning. A total of 143 participants aged 7–17, having relocated internationally with their working parent(s), recruited in local and international schools in Switzerland, were included in this study. Data were collected using an online survey after we collected consent. We assessed factors of adjustment using validated questionnaires: perceived stress and acculturative stress and the potential mediating roles of family functioning and resilience. We measured the outcome of adjustment through mental health difficulties and sociocultural adjustment. We used path analysis to test the model.

**Results:**

Results highlight the contributions of perceived stress and acculturative stress to TCK mental health and sociocultural adjustment. We also we found a mediation effect for resilience in the relationship between perceived stress and mental health. Family functioning was not a significant mediator in any relationship that we assessed.

**Discussion:**

We discuss implications for future research, promoting TCK adjustment and preventative psychotherapeutic interventions.

## Introduction

An international family relocation is both an opportunity and a challenge. Parents may hope that the experience will teach their children open-mindedness, flexibility, and world awareness. However, these benefits are inherently associated with the cost of losing friends and closeness to relatives and adjusting to new cultures, climates, and schools (Miyamoto and Kuhlman, 2001; McLachlan, 2007; Van Der Zee et al., 2007; Lijadi and Van Schalkwyk, 2017). For these children and adolescents, any benefits are conditional on their ability to buffer the stress associated with an international move (Vercruysse and Chandler, 1992; Pittmann and Bowen, 1994; Straffon, 2003; Van Der Zee et al., 2007; Weeks et al., 2010; Lucier-Greer et al., 2015; Sterle et al., 2018; McKeering et al., 2021). Children who relocate internationally with their working parents are labeled “Third Culture Kids” (TCK). The term, first introduced by Useem and Downie (1976), was later refined as “internationally mobile children and adolescents, who relocate with their families for work or advanced training purposes,” sometimes on short- to mid-term contracts, in this case, described as “highly mobile” (Pollock et al., 2010; Van Reken et al., 2017). The ability to adjust is central in the developmental trajectories of TCK and has implications for their mental health and social development. Research suggests that these individuals will develop a fluid and plural identity based on the hybrid integration of their cultural experiences (Bhabha, 2004; Jamshidian, 2019).

This study aims to strengthen our understanding of factors influencing TCK adjustment, by means of a heuristic model including primary factors and mediators. Haslberger et al. (2014) propose a comprehensive expatriate adjustment model, which considers the dimensions, domains, and dynamics of expatriate adjustment. The model acknowledges the interaction of dimensions of cognitive, affective, and behavioral indicators with various environmental and life domains in a dynamic, time-sensitive setting. Another model proposed by Naithani and Jha (2009) compiles adjustment factors identified throughout research in the field in a multidimensional model. This model isolates family, individual demographic and psychographic, work, and other environmental and contextual factors that influence expatriate adjustment. We assume that a network of personal and environmental factors will contribute to TCK adjustment (Nolen-Hoeksema and Watkins, 2011). However, empirically validated models of TCK adjustment, which account for developmental aspects of adjustment, still need to be included to the best of our knowledge.

**Figure 1:**
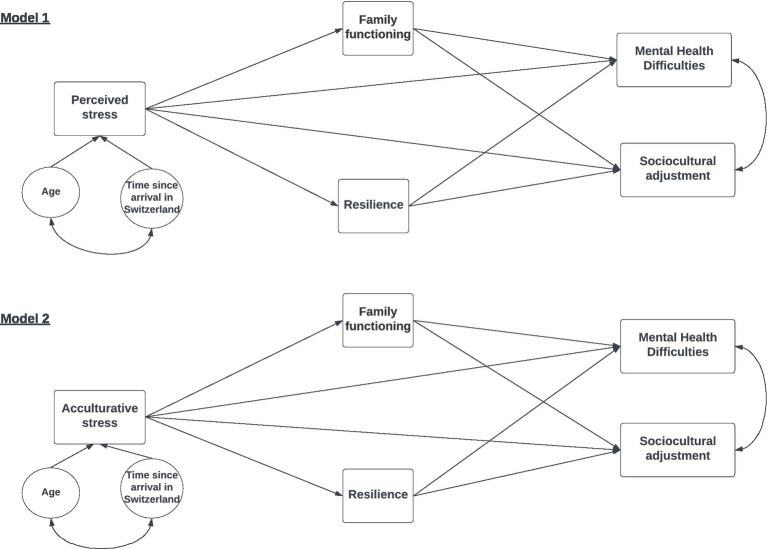
Hypothetical mediation models of TCK adjustment.

We chose the theoretical standpoint of transdiagnostic models such as Hierarchical Taxonomy of Psychopathology (HiTOP) and Research Domain Criteria (RDoC), which focus on the scaffolding of psychopathology, for the construction of our hypothesized model (Conway et al., 2012; Drury and Cuthbert, 2015; Kotov et al., 2017). As suggested within the RDoC initiative, this study considers the TCK sample as having encountered a particular stressful exposure and aimed to document the mechanisms involved in TCK adjustment. The HiTOP framework considers outcomes such as internalizing or externalizing symptoms as mental health indicators more accurately than bound clinical diagnoses. Ellis and colleagues consider developmental adjustment to stress a part of life’s ordinary course through their evolutionary–developmental perspective (Ellis et al., 2022). This evolutionary view of adjustment to change considers that the effects of adverse childhood experiences have an additive effect on developmental outcomes rather than the opposed cumulative risk view (Cicchetti and Tucker, 1994; Cicchetti and Rogosch, 1996; Felitti et al., 1998; Danese and McEwen, 2012). Stress and adversity in childhood will not always dysregulate or impair development but may regulate adaptive patterns of functioning (Belsky, 2008; Ellis and Giudice, 2014; Frankenhuis and Amir, 2022). The effectiveness of an adaptive pattern is determined by context, such that the analysis of any particular trait or behavior becomes inextricable from the context within which the pattern is expressed. In the case of TCK, the expression of adjustment will be read within the context of repeated sociocultural change, calling for a set of traits, skills, and resources that could be inefficient in a sedentary setting, in what is termed experience-driven plasticity (McLaughlin et al., 2014; Sheridan and McLaughlin, 2014). The threat and deprivation framework furthers our understanding of the adaptive response. For example, TCK might experience temporary loss of learning opportunities linked with language barriers or perceived threats due to unfamiliar surroundings or strains on parenting, all of which will participate in the shape of their developmental adaptive response. The threat and deprivation framework underlines that an adaptive response to the immediate environment may induce a ripple effect on responses to immediate and distal environments, perhaps influencing their susceptibility to future contexts and environmental strains (Ellis et al., 2022). A TCK who learns to cope with international relocation and reduce the stress linked with the immediate, unpredictable environment will benefit from learning and plasticity in developing neural pathways. This developmental adjustment may regulate future, less immediate responses in a TCK’s life, which emphasizes the importance of providing a framework for positive learning, resolution of stress, and understanding of the factors contributing to or hindering their adjustment (Cicchetti and Tucker, 1994). Hinde (1992) developed the idea that complex systems may be described without aiming to achieve a universal, exclusive, or uniform description of the system itself. With that in mind and informed by existing literature, we developed two TCK adjustment models (Figure 1).

Searle and Ward (1990) define adjustment as a dual concept comprising of psychological and sociocultural aspects, each conceptualized within different frameworks. On the one hand, psychological adjustment, framed within the stress and coping theoretical model, defines stress as “a particular relationship between the person and the environment that the person perceives as taxing or exceeding his or her resources and endangering his or her well-being” (Lazarus and Folkman, 1984, p. 19). Lazarus and Folkman’s model identifies perceived stress as a predictor of psychological adjustment: the perception of stress is retrieved from the actual stressor and is linked with an individual’s perception of their ability to cope and their assessment of the stressful situation. Such stressors include international relocation and the related unpredictability of a new environment, novelty, change associated with the new location, a new school, an unfamiliar language, and the necessity of fitting in and making new friends (Searle and Ward, 1990). The individual’s perception of stress can either ameliorate or hinder the adjustment process and, therefore, the individual’s mental health. On the other hand, sociocultural adjustment is explained through social learning theory and the acquisition of knowledge which facilitates integration within the new context, such as language skills or cultural norms (Bierwiaczonek and Waldzus, 2016). “Acculturation is the process of transferring culture from one group of people to another group in response to contact with one another” (Amason et al., 1999, p. 312). Sociocultural adjustment is a long-term outcome of that very process (Berry, 2006). While some research suggests that TCK may develop their own identity rather than assimilate into another culture, they are nonetheless confronted with changes upon transitioning from one country to another (Chankseliani, 2018). Acculturative stress results from acculturation and is described as a response to strains associated with changes in habits and patterns such as language, behaviors, interactions, and environment (Berry, 1980; Padilla et al., 1985). Acculturative stress in children relates to their feelings of being discriminated against or marginalized, negative feelings about being in a new country or speaking a new language, changes in cultures between schools, or changes in family dynamics due to local or cultural realities (Suarez-Morales et al., 2007; Morales, 2015). Symptoms that develop from acculturative stress may include internalizing and externalizing symptoms and family conflict and thus impact both social adjustment and mental health (Berry, 1988, 2006; Akhtar, 2012; Chien, 2013; Sirin et al., 2013).

Resilience is an adaptive process that enables an individual to function when confronted with risk factors that are expected to affect developmental and adjustment outcomes (Masten et al., 2012; Masten, 2014). Resilience has been extensively associated with better developmental outcomes in high-risk environments and is described as the ability to navigate and negotiate those challenges, potentially promoting mental health and well-being (Ong et al., 2006; Ahern et al., 2008; Ungar, 2010; DiCorcia and Tronick, 2011; Windle, 2011; Vieira et al., 2020). Many studies have identified the mediating role of resilience between life challenges and mental health outcomes and well-being in children and young adults (Fritz et al., 2018; Collazzoni et al., 2020; Tam et al., 2020; Ye et al., 2020; McLafferty et al., 2021).

Stress from a life change, such as international relocation, is managed by individual resources and social support (Wang et al., 2020). Family relationships are the first stage of a broader context that affects the child’s ability to regulate stress and navigate change. These relationships have a crucial influence on the outcomes of adversity during childhood. Research on how parenting affects a child’s ability to adjust is extensive and supports the idea that this is a factor that participates in differential sensitivity to stress and the longitudinal development of mental health (Chen et al., 2015; Cano et al., 2016; del Barrio et al., 2016; Lorenzo-Blanco et al., 2017; Keijser et al., 2020). In TCK, research shows the participating role of parenting on adjustment (Shaffer et al., 2001; Takeuchi et al., 2007; Haslberger and Brewster, 2008), although a recent review points to a gap in research on family adjustment in TCK (Sterle et al., 2018). Walsh proposes a framework that integrates family functioning as a transactional process that mediates adjustment in complex environmental situations through problem-solving, communication, and encouraging growth and learning (Walsh, 2016). Current research suggests that family resilience fosters individual resilience and improves adaptability (Hadfield and Ungar, 2018).

### Research goals

The current study aimed to examine the relationship between both perceived stress and acculturative stress and the two outcomes mental health difficulties and sociocultural adjustment, as well as the roles of resilience and family functioning in mediating these associations within a sample of TCK having relocated to Switzerland. Within each model, we considered the potential mediating role of resilience on the outcomes of adjustment: an intermediate process between the individual psychological processes and the outcomes of adjustment to the international relocation as a stress factor. Considering resilience as a mediator further enforces the viewpoint of developmental plasticity and adjustment through skill-building. The potential mediation of family functioning acknowledges the importance of context in development.

Our main aim was to assess the relationships in the hypothesized models using path analysis. The following research questions guided our study:

1. How do perceived and acculturative stress influence TCK adjustment (measured in terms of mental health difficulties and sociocultural adjustment)?
2. Is the relationship between the predictors of TCK stress and the outcomes of TCK adjustment mediated by (a) resilience and (b) family functioning?

Research question 1, hypothesized that increased perceived and acculturative stress will both be negatively associated with TCK mental health and with sociocultural adjustment. Regarding research question 2, we hypothesized that the expected associations between TCK stress predictors and TCK adjustment outcomes would all be mediated by resilience and family functioning.

## Methods

### Participants and procedures

This cross-sectional study is part of a more extensive longitudinal study, including broader measures of psycho-socio-cultural adjustment of TCK and their families over two-time points between 2017 and 2021 (Ooi et al., 2022). We recruited participants from international and local schools in Switzerland, multinational companies in Basel, and the city of Basel’s welcome comity. We distributed flyers and gave talks to approach target populations and invite them to participate. Inclusion criteria were (a) families with children between 7 and 17 years old, (b) parents relocating to Switzerland for employment, (c) both parent and child understanding and speaking English, and (d) neither parent nor child were Swiss citizens. The Ethics Committee of the Faculty of Psychology at the University of Basel issued an ethics approval for the study (Study No. 047–18-4). We obtained written informed consent and assent following the Declaration of Helsinki from participants and their parents prior to study-related procedures. All data were pseudo-coded without personal identifiers. We included and analyzed data from 143 child and adolescent participants in the present study.

### Measures

#### Outcomes of adjustment

We assessed mental health problems using the 25-item SDQ (Goodman, 1997). Parents rated their child/adolescent on a Likert-type scale ranging from 0 (*not true*) to 2 (*certainly true*). The sum of subscales of emotional problems, conduct problems, hyperactivity, and peer problems redeems a “total difficulties score.” The parent-rated version of the questionnaire has shown strong validity and predictive value for clinical disorders and is considered a good predictor of mental health in children and adolescents. The scale is used in many studies across numerous countries and cultures (Goodman et al., 2000; Bourdon et al., 2005). Higher scores indicate more internalizing and externalizing problems, suggesting more mental health problems. Items 7, 21, and 25 are reverse coded for the total difficulties score. Reliability for the total difficulties score was high (*α* = 0.80).

We assessed sociocultural adjustment using 21 items from the child version of the Sociocultural Adaptation Scale (SCAS-Child; Ward and Kennedy, 1999). Items explore behavioral and cognitive factors in sociocultural adjustments, such as making friends or feeling settled. We did not include the item “accepting/understanding the local political system,” assuming this item would not apply. Various scale versions exist, and it is easily adaptable to specific cultural settings. Parents of children below 12 completed the SCAS-Child on their behalf. Items are rated on a Likert-type scale ranging from 1 (*no difficulty*) to 5 (*extremely difficult*) and are summed to provide a total score, with higher scores indicating more difficulties, suggesting poorer sociocultural adjustment. The SCAS-Child has been widely used in previous studies and has shown good reliability and cross-sample consistency (Lowi et al., 2011; Yuan et al., 2013; Titzmann and Gniewosz, 2018). Internal consistency, as measured by Cronbach’s alpha, was 0.91.

#### Predictors

We assessed perceived stress using the 13-item Perceived Stress Scale for Children (PSS-C; White, 2014). Each child participant rated the items on a Likert-type scale ranging from 0 (*never*) to 3 (*a lot*). Items 2, 5, 6, 9, 10, 12, and 13 were reverse-coded, and the sum of all items provides a total score. The PSS-C has been widely used with culturally diverse samples and has shown good reliability and cross-sample consistency (Manns et al., 2021; Beranbaum et al., 2022; Takeuchi et al., 2022). Higher scores indicate higher levels of stress. Internal consistency, as measured by Cronbach’s alpha, was 0.82.

We assessed acculturative stress using the 4-item Acculturative Stress Inventory for Children (ASIC; Suarez-Morales et al., 2007). The scale measures participants rated items on a Likert-type scale ranging from 0 (*does not apply*) to 5 (*bothers me a lot*). Items explore perceived discrimination and immigration-related stress. Items are summed to provide a total score, with higher scores indicating higher levels of acculturative stress. The ASIC has been widely used with culturally diverse samples and has shown good reliability and cross-sample consistency (Thibeault et al., 2017; Schlaudt et al., 2021). Internal consistency, as measured by Cronbach’s alpha, was 0.66.

#### Mediators

We assessed resilience using the 12-item Child and Youth Resilience Measure (CYRM-12; Liebenberg et al., 2013). The scale has been used worldwide, and pilot studies have confirmed its validity and consistency across 11 countries and 14 communities (Renbarger et al., 2020). Each child participant rated the items based on a 3-point Likert scale ranging from 1 (*no*) to 3 (*yes*), and items are summed to provide a total score. Higher scores indicate higher levels of characteristics associated with resilience. As measured by Cronbach’s alpha, internal consistency was 0.64 and deemed acceptable (Nunnally and Bernstein, 1994; Pallant, 2001; Vaske, 2008).

We assessed family functioning using the 12 items that assess general functioning from the original 60-item McMaster Family Assessment Device (Epstein et al., 1983). Previous research suggests that the 12-item general functioning scale gives a good picture of family functioning (Ridenour et al., 1999; Georgiades et al., 2008; Al-Krenawi et al., 2009; Hosseini et al., 2018). Parents completed the 12 items using a Likert-type scale ranging from 1 (*strongly agree*) to 4 (*strongly disagree*). Odd items are reverse-scored, and all items are summed to provide a total score. Higher scores indicate poorer family functioning. The scale has satisfactory reliability, good test–retest reliability, and high validity and has been empirically tested across cultures in clinical and non-clinical settings (Epstein et al., 1983; Miller et al., 1985; Keitner et al., 1990; Morris, 1990; Wenniger et al., 1993). Internal consistency, as measured by Cronbach’s alpha, was 0.84.

#### Covariates

Based on the existing literature, we controlled for age (empirically divided into two groups, 7–12 years and 13–17 years) and the length of stay in Switzerland (in months) as these variables were shown to influence the adjustment outcome (Vercruysse and Chandler, 1992; Straffon, 2003; Ittel and Sisler, 2012).

### Data analysis

Research data were analyzed using R (Version:4.02). (The R Foundation for Statistical Computing for Windows; Version 3.5.1 for Windows^1^). We used univariate statistics (mean, standard deviation, and graphical displays) to describe the participants’ demographics and study variables. Then, we performed correlation analyses to examine the bivariate associations among the variables of interest. We checked for multivariate normality, multivariate outliers, and multicollinearity, as recommended by Kline (2015). No cases had to be removed from the dataset and multicollinearity was no issue. Models were kept separate because of a suppression effect between acculturative stress and perceived stress. A manipulation check showed that participants all completed the survey within an acceptable time frame (Curran, 2016).

We conducted the path analyses using the R package Lavaan (Rossee, 2012; Li, 2016). Since the model was just identified (c.f. Figure 1), both models fitted the data perfectly. Hence, no assessment of goodness of fit was necessary (i.e., Comparative Fit Index, CFI, Tucker-Lewis Index, and TLI were equal to 1). Regarding path coefficients, we provide unstandardized estimates and confidence intervals from bootstrapping from 500 samples, including the corresponding *z*-statistics and *p*-values, plus the standardized estimates. We assessed direct, indirect, and total effects. When the total effect was significant, then the direct and indirect effects were used to determine if partial mediation had occurred, and, in case the direct effect was not significant, full mediation was assumed (Preacher and Hayes, 2004; Zhao et al., 2010; Gunzler et al., 2013). When indirect effects were significant, we accepted that mediation had occurred (Preacher and Hayes, 2004; Zhao et al., 2010; Gunzler et al., 2013). Estimates of standard errors were based on the Delta method.

## Results

### Sample description

The total number of children who participated in this study was 143. We dealt with missing values using the full information maximum likelihood method, thereby assuming a missing at random (MAR) pattern *sensu* Rubin (Rubin, 1976). Parents of 17 participants did not complete the demographic section, although their children answered the questionnaires. Demographics are hence based upon 126 observations and 17 missing values. Among the child participants, 76 (60%) were females, and 50 were male (40%). Participants ages were evenly distributed between both groups: 51 (40%) were below 13 years, and 75 (60%) were aged from 13 to 17. The average time spent in Switzerland was 53.3 months (ranging from 51 to 66 months). The child’s ethnicity was European/Caucasian in 89 cases (71%), Asian in 12 cases (10%), multiple in eight cases (6%), Hispanic in four cases (3%), and other in 13 cases (10%) (including North American, South American, and other than specified). Participant children were of 55 different nationalities, including 23 combined nationalities. Parents reported English as the first language spoken at home in 82 (65%) cases, whereas German was the first language in two cases, Italian in three cases, and French in one case. German, French, and Italian are the three official languages of Switzerland.

### Descriptive statistics

Table 1 presents the means and standard deviations for each variable involved in the two models, except for covariates, and the correlation matrix among these variables.

**Table 1:** Descriptives and correlations of study variables.

Variable	*N*	Mean (SD)	1	2	3	4	5	6
1. Perceived stress: PSS-C	138	149.9 (6.69)	1					
2. Acculturative stress: ASIC	138	7.75 (4.08)	0.42*	1				
3. Family functioning: MMFF	126	19.33 (4.57)	0.04	0.02	1			
4. Resilience: CYRM-C	122	30.98 (3.18)	−0.56**	−0.13	−0.09	1		
5. Mental health difficulties: SDQ	125	7.05 (5.17)	0.12	−0.1	0.28	−0.33*	1	
6. Sociocultural adaptation: SCAS	95	44.61 (12.14)	0.52**	0.47*	0.02	−0.36*	>0.02	1

N, number of observations; SD, standard deviation; PSS, perceived stress scale for children; ASIC, acculturative stress inventory for children; MMFF, McMaster family assessment device; SDQ, strengths and difficulties questionnaire, total difficulties score; SCAS, sociocultural adaptation scale – child version. *p < 0.05, **p < 0.01.

Largest associations were observed between perceived stress and resilience and between perceived stress and sociocultural adaptation. Somewhat weaker but still considerable associations were further found between acculturative stress and sociocultural adaptation, between resilience and mental health difficulties, and between resilience and sociocultural adaptation. Note also the relatively high correlation between perceived and acculturative stress.

Table 2 presents the regression statistics for each model. As shown in this table, perceived stress, resilience and sociocultural adjustment each account for >30% of the variance explained in model 1. Family functioning has low incidence score for both models. In both models, sociocultural adjustment accounts for >10% of variance than mental health difficulties.

**Table 2:** Regression statistics.

*R* ^2^	Model 1	Model 2
PSS	0.35	
ASIC		0.14
MMFF	0.002	0.001
CYRM	0.31	0.01
SDQ	0.18	0.19
SCAS	0.33	0.36

*N* = 143, PSS, perceived stress scale (child version); ASIC, acculturative stress inventory for children; CYRM, child and youth resilience measure; MMFAD, Mc Master family assessment device; SCAS, sociocultural assessment scale (child version); SDQ, strengths and difficulties questionnaire total difficulties score.

**Figure 2:**
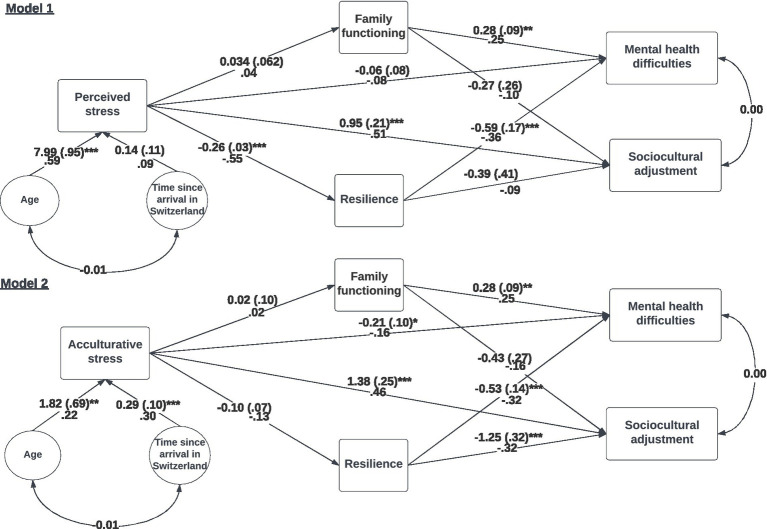
Path models of the hypothetical models of TCK adjustment. The models show the coefficients of the direct paths from the path analysis. The coefficients above lines are Beta coefficients (Standard error). Straight lines show the effects tested in the regression analysis. Curved lines show correlations. The standardized solution coefficient (effect size) is below the line. *p < 0.05, **p < 0.01, ***p < 0.001.

Path coefficients are shown in Figure 2. Table 2 summarizes the results of direct, indirect, and total effects in each model.

#### Model 1

As shown in Table 3, one of the two total effects was significant (research question 1). Thus, perceived stress was positively associated with sociocultural adjustment difficulties, with a large effect size. Concerning the mediating role of resilience and family functioning in the relationship between perceived stress and the outcomes of TCK adjustment (research question 2), we found that the relationship between perceived stress and mental health difficulties was mediated by resilience (*b* = 0.15, *p* < 0.05*, see Table 3). This mediation effect can be considered an indirect only effect, as the corresponding direct effect between perceived stress and mental health was not significant (Table 3), suggesting full mediation and the unlikelihood of an omitted mediator (Zhao et al., 2010). The effect size of this mediation effect was small to medium.

**Table 3:** Defined parameters showing measured effects from each model are presented in Figure 2.

Model	Effect type	Path	Path coefficient (CI lower/upper)		Standard error	*Z*-value	*P* (>Z)	Standardized path coefficient
Model 1								
	Total effects	PSS-SDQ	0.097 (−0.064/0.249)		0.070	1.385	0.166	0.125
		PSS-SCAS	1.048 (0.724/1.333)		0.175	5.997	<0.001***	0.561
	Direct effects	PSS-SDQ	−0.069 (−0.246/0.082)		0.080	−0.865	0.387	−0.089
		PSS-SCAS	0.953 (0.540/1.365)		0.216	4.406	<0.001***	0.511
	Indirect effects	PSS-MMFF-SDQ	0.01 (−0.024/0.051)		0.018	0.532	0.595	0.012
		PSS-MMFF-SCAS	−0.009 (−0.138/0.024)		0.020	−0.472	0.637	−0.005
		PSS-CYRM-SDQ	0.157 (0.057/0.276)		0.050	3.154	0.002*	0.202
		PSS-CYRM-SCAS	0.103 (−0.132/0.310)		0.109	0.947	0.344	0.055
Model 2								
	Total effects	ASIC-SDQ	−0.151 −0.387	0.090	0.115	−1.32	0.187	−0.119
		ASIC-SCAS	1.504 0.951	1.986	0.263	5.718	<0.001***	0.5
	Direct effects	ASIC-SDQ	−0.214 −0.433	0.000	0.107	−2.008	0.045*	−0.169
		ASIC-SCAS	1.388 0.845	1.907	0.254	5.459	<0.001***	0.461
	Indirect effects	ASIC-MMFF-SDQ	0.008 −0.060	0.082	0.029	0.286	0.775	0.007
		ASIC-MMFF-SCAS	−0.013 −0.188	0.098	0.045	−0.278	0.781	−0.004
		ASIC-CYRM-SDQ	0.055 −0.001	0.137	0.040	1.366	0.172	0.043
		ASIC-CYRM-SCAS	0.128 −0.026	0.342	0.093	1.379	0.168	0.043

*N* = 143, *p < 0.05, **p < 0.01, ***p < 0.001, CI, confidence interval from bootstrapping; PSS, perceived stress scale (child version); ASIC, acculturative stress inventory for children; CYRM, child and youth resilience measure; MMFAD, Mc Master family assessment device; SCAS, sociocultural assessment scale (child version); SDQ, strengths and difficulties questionnaire total difficulties score. Effect sizes for standardized path coefficients: >0.1 = small, >0.3 = medium, >0.5 = large.

The remaining three indirect effects did not significantly differ from zero. Thus, both indirect effects, with family functioning as a mediator, were unimportant. In this context, it is of note that all path coefficients pointing to or leading away from the mediator family functioning were not significantly different from 0 except for the coefficient between family functioning and mental health difficulties. This highlights a correlation in between family functioning and mental health difficulties outside of the relationship with the predictor perceived stress (see Figure 2).

We found a significant effect of age on perceived stress (see Figure 2). Time, however, did not influence perceived stress.

#### Model 2

As shown in Table 3, one of the two total effects was significant (research question 1). Thus, acculturative stress was positively associated with sociocultural adjustment difficulties, with a large effect size. We found that only the direct path from acculturative stress to mental health difficulties was significant. In contrast, the total effect was not significant, suggesting there may be one or more other relevant mediators for this association (Zhao et al., 2010).

Concerning the mediating role of resilience and family functioning in the relationship between the predictors of acculturative stress and the outcomes of TCK adjustment (research question 2), we found that all four indirect effects were unimportant. Consequently, neither family functioning nor resilience mediated the relationship between acculturative stress and mental health difficulties or sociocultural adaptation.

## Discussion

The objective of this exploratory study was to determine pathways of adjustment in TCK based on two hypothesized models of TCK adjustment. Our findings contribute to enriching our understanding of the process of adjustment in TCK by providing evidence toward an intricate network of factors involved in psychological and sociocultural adjustment. The conceptual separation between adjustment types as proposed by Searle and Ward (1990) is justified in our findings. However it seems that these distinctive outcomes may be expressions of underlying intrinsic and extrinsic characteristics: the findings in this study emphasize that the personal experience of stress could be a key indicator in adjustment outcomes for TCK: intrinsic personal variables of cognitive interpretations of a stressful situation (perceived stress) and sensitivity to cultural change and discrimination (acculturative stress) are founding processes for mental health and adjustment to relocation in our sampled TCK. More specifically, our study provides evidence of the influence of perceived stress on sociocultural adjustment and, mediated by resilience, on mental health difficulties. Our second model shows acculturative stress’s influence on adjustment outcomes: sociocultural adjustment and mental health difficulties. In this second model, no mediation is found, despite significant pathways between family functioning and mental health difficulties for one and resilience on both outcomes of adjustment for the other. This finding is in line with work by Krueger (1999, 2002) and Helzer and Hudziak (2002) on comorbidity and the structure of mental disorders; theories of psychopathology, which stem from Kruger’s work, suggest feedback associations between latent psychological variables and transdiagnostic outcomes (Liu and Alloy, 2010; Conway et al., 2012). Likewise, we find that different subtypes of stress influence various outcomes, with the potential to generate feedback on stress due to adjustment difficulties. Moreover, our models’ results suggest another mediator’s existence, which underlines the system’s complexity.

Another finding from our study is the mediated relationship between perceived stress and mental health through resilience. Specifically, we found that increased levels of perceived stress led to decreased resilience, which in turn, led to more mental health difficulties. Our finding is in line with previous findings on the mediating role of resilience between life challenges and mental health outcomes (Fritz et al., 2018; Tam et al., 2020; Ye et al., 2020; McLafferty et al., 2021). On the other hand, family functioning affects mental health, although this variable does not mediate stress. Our sample’s family functioning difficulty scores are slightly lower than the normal range reported for a non-clinical sample (Byles et al., 1988; Hogye et al., 2022). According to this measure, families communicate well about the move and provide a safe space for TCK, which aligns with previous findings (Selmer and Lam, 2004). Previous research suggests that after an international relocation, other family members are also disrupted in their habits; the international assignment may inverse parental roles, and all family members may experience increased stress and disrupted quality of life (Selmer and Lam, 2004; van der Zee et al., 2007). We assume that such family disruptions may affect family functioning and therefore TCK mental health, if closer-knit family relations do not compensate for increased family stress or personal struggles. Future studies could refine our understanding of the role of parenting strategies and parental satisfaction on TCK adjustment (Bornstein et al., 2018).

In summary, our findings from a healthy sample of TCK support the evolutionary theory which suggests that personal characteristics forge an individual’s ability to cope with life stress (Ungar, 2008; Carver and Connor-Smith, 2010; Maranges and Reynolds, 2020; Maranges and Strickhouser, 2021). Prevention programs focusing on stress (perceived and/or acculturative stress) for children and teens relocating internationally seem fit to decrease cognitive vulnerability and improve adjustment and overall outcomes (Gillham et al., 2012). Skill sets to oppose stress could include mindfulness-based stress reduction techniques, psychological flexibility, optimism, coping skills, social support networks, physical activity, and a personal moral compass (Kashdan and Rottenberg, 2010; Fritz et al., 2018; Iacoviello and Charney, 2020; Tam et al., 2020; Arslan and Allen, 2021).

In addition, our results underline the role of resilience as an “intervening variable” on the outcome of mental health. Individuals who are resilient in their ability to cognize stress, change, and contrasts will adjust better (Hampel and Petermann, 2006). This intervention supports the assertion that resilience-building programs could benefit mental health and, more generally, adjustment in TCK (Waugh and Koster, 2015; Collazzoni et al., 2020). Resilience enhancement programs associated with stress reduction techniques have been effective in various populations (Black, 1993; Kim et al., 2018). Moreover, in the case of TCK having developed a mental health disorder and understanding that these were not better explained by any other tangible trigger, treatment programs could include stress reduction and resilience-building skills (Van Daele et al., 2011; Sharma and Rush, 2014; Joyce et al., 2018).

### Limitations and directions for future studies

This study has several limitations. Firstly, we conducted the study during the first and second waves of the COVID-19 pandemic. International border closures and uncertainties, as well as social isolation, will have triggered a significant increase in stress for TCK and their families, mainly because of the physical impossibility of reaching their homeland and relatives and friends in their home countries. Research shows that stress from the COVID-19 pandemic hinders adolescent adjustment (Ellis et al., 2020; Ye et al., 2020). It is only possible to report the distinction between COVID-19-related perceived stress and that more closely related to the relocation, although there may be a connection between the two. However, our measure of acculturative stress is specific to the relocation situation. Replication studies would allow a further investigation of our findings post-pandemic and account for the low reliability of ASIC and CYRM found in this study. Mixed methods studies, including qualitative interviews, could help determine which stress factors are essential in perceived and acculturative stress.

Second, this study did not integrate longitudinal adjustment measures due to high dropout rates and recruitment difficulties related to the COVID-19 pandemic. Longitudinal measures of adjustment constitute an interesting insight into the dynamics of the adjustment process. They should be considered for further studies, as suggested by our study’s correlation between acculturative stress and length of stay in Switzerland.

Also, our sample experienced slight contextual variation (same country, same period) and shared many demographic similarities (socio-economic status, location, attended school). We assume that the measured variation in mental health and sociocultural adjustment was due to the measured processes, although we cannot rule out other contributions to TCK adjustment.

This exploratory study focused on particular traits in a defined ecological sample. Our findings are specific to this population and withhold an emphasis on sociocultural change, which could be replaced in other studies to reproduce our results and account for variance in alternative clusters of developing children who share alternative similarities (i.e., migrant children or children undergoing a divorce). Future studies could also include comparison groups between clinical and non-clinical samples or TCK versus non-TCK specific samples.

Future research on TCK could include exploring the role of developmental stages and locus of control in perceived stress (Boyraz et al., 2019; Srivastava and Kapoor, 2021). As found in the correlation between age and perceived stress in our study, the ability to process the disruptions from a move depends on a child’s cognitive, emotional and social developmental stage (Vercruysse and Chandler, 1992; Ittel and Sisler, 2012). Moreover, a subtle difference in status by which their relocation is an independent life event (fateful). In contrast, it may be considered a dependent life event for their parents, who chose to relocate. This element of choice makes TCK’s cognitions about and around the move particularly sensitive.

## Conclusion

The findings from this study contribute to a better understanding of the processes involved in TCK adjustment, particularly the roles of perceived stress and acculturative stress on mental health and sociocultural adjustment, respectively as well as the mediating role of resilience and family functioning. As such, this study expands on our knowledge of the process of TCK adjustment and offers a pragmatic measure of the dual process described by Ward and Kennedy (1999).

Our findings contribute to building evidence of the existence of specific personal sensitivities to stress exposure (Hammen, 2006; Conway et al., 2012). Our study is an example of the pathways toward the ability to adjust to life stress and offers an insight into the etiology of potential chronic difficulties in adjustment and sensitivities to the development of psychopathology.

We propose that building specific skills such as resilience and stress reduction strategies would promote developmental plasticity and conditional adjustment to family relocation stress such that TCK may develop an adaptive function that they could exert to thrive in an ever-changing world (Ellis et al., 2017; Ellis and Del Giudice, 2019).

## Data availability statement

The datasets presented in this study can be found in online repositories. The names of the repository/repositories and accession number(s) can be found at: Jones et al. (2022). Data set – Stress, Mental Health and Sociocultural Adjustment in Third Culture Kids: The Mediating Roles of Resilience and Family Functioning [Data set], Zenodo, https://doi.org/10.5281/zenodo.7304328.

## Ethics statement

The studies involving human participants were reviewed and approved by The Ethics Committee of the Faculty of Psychology at the University of Basel issued an ethics approval for the study (Study No. 047-18-4). Written informed consent to participate in this study was provided by the participants’ legal guardian/next of kin.

## Author contributions

EJ, YO, MR, and JG contributed to the study concept and design. EJ and AM analyzed the data. EJ drafted the manuscript. JG, MR, YO, and AM contributed to critical advice and revisions of the manuscript. All authors contributed to the article and approved the submitted version.

## Conflict of interest

The authors declare that the research was conducted in the absence of any commercial or financial relationships that could be construed as a potential conflict of interest.

## Publisher’s note

All claims expressed in this article are solely those of the authors and do not necessarily represent those of their affiliated organizations, or those of the publisher, the editors and the reviewers. Any product that may be evaluated in this article, or claim that may be made by its manufacturer, is not guaranteed or endorsed by the publisher.

## Footnotes

^1^ www.rproject.org
